# The Metabolic Response of Various Cell Lines to Microtubule-Driven Uptake of Lipid- and Polymer-Coated Layer-by-Layer Microcarriers

**DOI:** 10.3390/pharmaceutics13091441

**Published:** 2021-09-10

**Authors:** Claudia Claus, Robert Fritz, Erik Schilling, Uta Reibetanz

**Affiliations:** 1 Institute of Medical Microbiology and Virology, Faculty of Medicine, University of Leipzig, Johannisallee 30, 04103 Leipzig, Germany; 2 Institute for Medical Physics and Biophysics, Faculty of Medicine, University of Leipzig, Härtelstrasse 16-18, 04107 Leipzig, Germany; fritz_robert@gmx.net; 3 Institute of Clinical Immunology, Faculty of Medicine, University of Leipzig, Johannisallee 30, 04103 Leipzig, Germany; erik.schilling@medizin.uni-leipzig.de

**Keywords:** supported lipid bilayer, polymer, layer-by-layer, drug delivery system, uptake, intracellular transport, cell metabolism, immunofluorescence

## Abstract

Lipid structures, such as liposomes or micelles, are of high interest as an approach to support the transport and delivery of active agents as a drug delivery system. However, there are many open questions regarding their uptake and impact on cellular metabolism. In this study, lipid structures were assembled as a supported lipid bilayer on top of biopolymer-coated microcarriers based on the Layer-by-Layer assembly strategy. The functionalized microcarriers were then applied to various human and animal cell lines in addition to primary human macrophages (MΦ). Here, their influence on cellular metabolism and their intracellular localization were detected by extracellular flux analysis and immunofluorescence analysis, respectively. The impact of microcarriers on metabolic parameters was in most cell types rather low. However, lipid bilayer-supported microcarriers induced a decrease in oxygen consumption rate (OCR, indicative for mitochondrial respiration) and extracellular acidification rate (ECAR, indicative for glycolysis) in Vero cells. Additionally, in Vero cells lipid bilayer microcarriers showed a more pronounced association with microtubule filaments than polymer-coated microcarrier. Furthermore, they localized to a perinuclear region and induced nuclei with some deformations at a higher rate than unfunctionalized carriers. This association was reduced through the application of the microtubule polymerization inhibitor nocodazole. Thus, the effect of respective lipid structures as a drug delivery system on cells has to be considered in the context of the respective target cell, but in general can be regarded as rather low.

## 1. Introduction

The research topic of smart nanocarriers has gained much attention since their successful application as a delivery system for mRNA vaccines, including the SARS-CoV-2 spike protein [1,2]. Additionally, their application in cancer immunotherapeutics for the transport of immune modulators is of high interest as the activation of a humoral or cellular immune response could support recognition and eventually killing of tumour cells, and as such the elimination of the entire tumour [3,4,5]. In this context, artificial lipids as part of functionalized liposomes and micelles or as a supported bilayer on top of other drug delivery systems are of special interest. Besides the high drug loading efficiency of such lipid structures, general characteristics such as an easy to achieve surface functionalisation ensure prolonged blood circulation, high cellular uptake and low toxicity [6,7].

Uptake depends on many parameters such as size, shape, surface charge and surface modification or functionalization of the LbL microcarriers and cell type and morphology, which is reflected by various uptake routes for LbL microcarriers including caveolae-mediated endocytosis as well as macropinocytosis and phagocytosis [8,9]. However, the intracellular transport route of polymer and lipid-based microcarriers, and in this regard their impact on cellular metabolism, are still, in most parts, unclear [10]. In this context, the importance of cellular metabolism is two-fold. First, an alteration in the metabolic state of the target cell would point toward a modulation of cellular functions by the carrier and potential off-target effects that could occur besides effects by the delivered compound. Second, endocytotic processes are energy-dependent processes [11]. The release of these microcarriers from endosomes is characterised by a time-delay, which also depends on several parameters such as microcarrier size, material, charge, stiffness or respective functionalisation [12,13,14]. This in turn could reduce the availability of endosomes for cellular homeostasis and induce metabolic countermeasures in the target cell.

Thus, this study was initiated to investigate whether lipid-based microcarriers affect cellular metabolism and differ from microcarriers lacking such a bilayer. To address metabolic alterations by lipid-based surface modifications, a supported lipid bilayer (SLB) was assembled onto a layer-by-layer biopolymer-coated microcarrier. Such a micrometer-sized system has two advantages: At first, it allows for an accurate and easy to follow-up application of a defined number of microcarriers within the cell through the addition of fluorescence markers. Moreover, intracellular fluorescence tracing of microcarriers could be combined with immunofluorescence staining of cellular markers [15]. This methodological approach enables assessment of the association of microcarriers with cellular compartments. At second, microcarriers are in vitro already well-established drug delivery systems, in particular for local administrations [16,17,18,19]. Moreover, the modular construction by stepwise assembly of biopolymers onto a solid core with subsequent core dissolution facilitates a multifunctional and highly biocompatible design [20,21]. Active agents can be assembled either into the core or the multilayer. Additionally, functional components, such as surface-assembled peptides or antibodies can be independently added, which allows for control of the time-point of drug release and of the amount of the delivered drugs [22,23,24].

Here, a protamine sulphate and dextran sulphate biopolymer multilayer on a microcarrier template was covered with a bilayer composed of phosphoserine/phosphocholine at a 1:1 molar ratio and with a biotinylated lipid anchor that allows for further functionalization in future applications [25,26]. As a control design, dextran sulphate as a negatively charged polymer was used as an outer layer. The comparison of both microcarrier types allowed for assessment of lipid bilayer-associated effects on the respective target cell. Their impact on central cellular metabolic pathways as an important readout for energy homeostasis was studied in permanent human (A549 and human embryonic kidney HEK293T/17 cells) and animal cell lines (mouse 3T3.CD4.CXCR4 and Vero African green monkey kidney cells) besides primary human macrophages MΦ. These cell types were selected based on various criteria. First, Vero, HEK293T/17 and A549 cells are already characterized in different microcarrier studies [10,11,13,24]. Second, they display a different metabolic background, e.g., the glycolytic tumour cell line A549 in comparison to HEK293T/17 with a high rate of aerobic respiration. Third, these cell types represent different options for the study of drug-delivery by microcarriers in the context of its application as a drug delivery tool. Mouse 3T3.CD4.CXC4 provide insights in their application in mouse models. Vero cells are a well-characterised cell line for various applications, including virus infection studies, toxin assays and the production of vaccines [27]. Human primary MΦ represent an interesting target cell type for immunotherapeutic applications. Extracellular flux analysis by the Agilent Seahorse XFp Analyzer allows for a direct measurement of the parameters oxygen consumption rate (OCR) for mitochondrial respiration and extracellular acidification rate (ECAR) for glycolysis. Besides the cellular metabolic phenotype in response to the uptake of the different microcarriers by the cell lines used in our study, uptake rate and intracellular localization of the microcarriers were addressed. Our previous work [15] already identified the association of microcarriers with microtubule filaments in Vero cells. Thus, we focused on intracellular distribution of microcarriers in relation to microtubules in the cell lines included in our investigations.

In general, the influence of microcarriers on cellular metabolism was rather low. However, microcarriers with a SLB revealed in Vero cells a decrease in metabolic potential as compared to cells with microcarriers lacking a lipid bilayer and cells without microcarrier application. On this cell line the association of SLB-supported microcarriers with microtubules was increased. Additionally, microcarriers equipped with an SLB showed in most of the analysed cell lines a higher association with microtubule filaments. Moreover, a localisation in close proximity to the nucleus lead to its visible deformation, which was also more profound for SLB microcarriers. Despite the micrometre-sized diameter (5.7 µm), microcarriers appear to have a low impact on cellular metabolism and thus on cellular functions. This observation supports their use as a drug delivery system (DDS) for local administration and highlights their potential use for targeted transport of agents to the nucleus.

## 2. Materials and Methods

### 2.1. Reagents

Nocodazole was purchased from Sigma Aldrich (Taufkirchen, Germany), diluted in DMSO (Sigma Aldrich) as a 5 mg/mL stock solution and used in final concentration of 10 µM per well.

### 2.2. Cultivation of Permanent Cell Lines

All permanent cell lines used in this study, namely A549 (ATCC^®^ CCL-185™, adenocarcinomic alveolar basal cell line), HEK (HEK-293T/17, ATCC^®^ CRL-11268™, epithelial human embryonic kidney cell line), 3T3 (NIH-3T3.CD4.CXCR4, fibroblast mouse cell line, Dr. Dan R. Littman, Skirball Institute of Biomolecular Medicine and Howard Hughes Medical Institute New York University Medical Center, New York, NY, USA), and Vero cells (ATCC CCL-81, African green monkey kidney epithelial cell line) were cultured in Dulbecco’s modified Eagle’s medium (DMEM, Thermo Fisher Scientific, Schwerte, Germany) supplemented with 10% foetal calf serum (FCS, Invitrogen Life Technologies/Thermo Fisher Scientific) and 100 U/mL penicillin and 100 mg/mL streptomycin (Thermo Fisher Scientific).

### 2.3. Preparation of Human MΦ

Human peripheral blood derived mononuclear cells were isolated from buffy coats of healthy blood donors (supplied by Institute of Transfusion Medicine University Hospital Leipzig, Leipzig, Germany) The JE-5.0 elutriation system (Beckman Coulter, Brea, CA, USA) was used for isolation of human monocytes by counter-flow elutriation as described previously [28]. Purity of monocytes was determined by flow cytometry through application of the APC anti-human CD14 antibody (M5E2, BioLegend, San Diego, CA, USA). Monocytes with a purity of at least 90% were suspended in RPMI 1640 medium supplemented with 10% (*v*/*v*) FCS, 100 U/mL penicillin and 100 mg/mL streptomycin at a density of 5 × 10^5^ cells/mL. For their differentiation in MΦ, 500 IU/mL recombinant human granulocyte-macrophage colony stimulating factor (GM-CSF, LEUKINE^®^ sargramostim, Genzyme, Neu-Isenburg, Germany) was added to Teflon bags (Zell-Kontakt, Nörte-Hardenberg, Germany) followed by an incubation period of 7 d. This in vitro generated MΦ type resembles M1 MΦ and as such is referred in our manuscript to a M1-like differentiation state (M1-like MΦ or GM-MΦ). Experiments with M1-like MΦ as outlined in this study were approved by the ethics committee at the Medical Faculty of the University of Leipzig under ethics license 001/19-ek.

### 2.4. Layer-by-Layer Coating of Microcarriers

5.70 μm ( ± 0.2 μm) SiO_2_ particles (microParticles GmbH, Berlin, Germany) were used as a template to be alternatingly coated with protamine sulfate (PRM, MW ~4 kDa, Sigma Aldrich) and dextran sulfate (DXS, MW 40 kDa, Sigma Aldrich) to form a biopolymer multilayer. As a stable internal layer design for intracellular detection, synthetic polymers poly(allylamine-hydrochloride) (PAH, 56 kDa, Sigma Aldrich) and polystyrene sulfonate (PSS, MW 70 kDa, Sigma Aldrich) were used resulting in the following final coating pattern: SiO_2_: [PAH/PSS]_2_ [PAH/DXS] [PRM/DXS]. For coating, 2 mg/mL PRM and DXS were dissolved in 0.1 M NaCl solution and 2 mg/mL PAH and PSS in 0.5 M NaCl. Microparticles were transferred to the respective polymer solution and under vigorous shaking for 10 min. After centrifugation, the pellet was resuspended in 0.1 M NaCl solution, washed, and centrifuged again. Washing was repeated three times.

For special applications, PAH was labelled with rhodamine isothiocyanate (RITC, Sigma Aldrich) for fluorescence detection. Here, PAH was dissolved in borate buffer (pH 8.5) and mixed with methanol at an equal ratio. RITC was dissolved in methanol and slowly added to the PAH solution under constant mixing. After two days of stirring, the sample was dialysed in double distilled water to remove the buffer and was subsequently solvent extracted after mixing with butanol. PAH-RITC, dissolved in the water phase, was separated from RITC and then collected and freeze-dried for storage. Using labelled PAH, the previous coating pattern changed to SiO_2_: [PAH/PSS] [PAH-RITC/PSS] [PAH-RITC/DXS] [PRM/DXS].

### 2.5. Assembly of a Supported Lipid Bilayer (SLB)

Based on the assembly procedure as described in detail in Goese et al. [25], phosphatidylserine (POPS, 784 Da, 25 mg/mL, Avanti Polar Lipids, Alabaster, AL, USA) and phosphatidylcholine (POPC, 760 Da, 25 mg/mL, Avanti Polar Lipids, Alabaster, AL, USA) were mixed at equal molar ratios with 0.5 mol% (DS)PE-PEG(2000)-Biotin (Avanti Polar Lipids, Alabaster, AL, USA). Resuspended in PBS and heated at 37 °C, this mixture was extruded 19 times to form 50 nm liposomes by using a polycarbonate membrane (hole diameter = 50 nm, Avestin, Ottawa, IL, USA). Before the SLB was assembled, a final outer PRM layer was added to the biopolymer coating of the microcarriers. The liposomes were then co-incubated with the microcarriers at 37 °C and under constant shaking followed by three washing steps in PBS. The liposomes adsorb onto the surface of the positively charged outermost layer of the microcarriers and fuse to build up a homogeneous supported lipid bilayer. The following coating pattern was generated: SiO_2_: [PAH/PSS] [PAH-RITC/PSS] [PAH-RITC/DXS] [PRM/DXS] [PRM/SLB].

### 2.6. Application of Microcarriers to Cells

Primary M1-like MΦ were plated on glass coverslips at a density of 2.5 × 10^5^ cells per well of a 24 well plate (Greiner Bio-One, Frickenhausen, Germany, followed by an adherence phase of 2 h before addition of microcarriers. Permanent cell lines A549, 3T3 and Vero were plated at a density of 1 × 10^5^ cells per well, while HEK cells were plated at 2 × 10^5^ cells per well. Followed by an incubation period of 24 h microcarriers were added. For all experiments, microcarriers were applied at a ratio of 2:1 (microcarriers:cells). This was followed by an additional incubation time of 16 h for all cell types employed. Additionally, Vero cells were also analysed after an incubation period of 6 h.

### 2.7. Assessment of Mitochondrial Respiration and Glycolysis through Extracellular Flux Analysis

Extracellular flux analysis allows for real-time measurement of metabolic activity in living cells: the oxygen consumption rate (OCR) refers to mitochondrial respiration and extracellular acidification rate (ECAR) reveals changes in the pH in association with glycolytic activity [29]. Plating of cells into the XFp miniplate was done with 2 × 10^4^ cells per well for A549 and 3T3 cells, 1.5 × 10^4^ cells per well for Vero cells, and 4 × 10^4^ cells per well for HEK and MΦ. The experiment was repeated three times (*n* = 3, biological replicates) and measured as duplicated (*n* = 2, technical replicates) per experiment. Extracellular flux analysis was performed on the XFp flux analyser (Agilent Seahorse Technologies, Santa Clara, CA, USA) with the Agilent Seahorse cell energy phenotype test kit, which enables characterization of the metabolic phenotype through co-injection of the ATPase inhibitor oligomycin (1 µM for all cell lines) and trifluoromethoxy carbonylcyanide phenylhydrazone (FCCP, 0.8 µM for A549, 3T3 and Vero, 0.6 µM for HEK and 1.2 µM for MΦ). The ratio of baseline (basal) OCR and ECAR (OCR/ECAR ratio) obtained at measurement point three was used to compare the metabolic phenotype between different cells types [30]. For normalization of all permanent cell lines, Bradford assay was applied. Primary M1-like MΦ are non-dividing and comparison between samples was based on equal cell numbers during plating.

### 2.8. Immunofluorescence Analysis and Application of the Mito Tracker Dye

Immunofluorescence staining was applied according to Scheffler et al. [15] Anti-α-tubulin primary antibody (Sigma Aldrich) was applied to PFA-fixed cells at 1:400 dilution in PBS for 60 min at 37 °C. Subsequently, donkey IgG anti-mouse IgG (H + L) cross-absorbed (min X bovine, chicken, goat, guinea pig, horse, hamster, human, rabbit, sheep) Alexa Fluor 488 was added as secondary antibody at a 1:100 dilution in PBS for 45 min at 37 °C. Samples were mounted with Fluoromount G (Invitrogen Life Technologies/Thermo Fisher Scientific) containing DAPI.

MitoTracker Red CMXRos (MitoTracker, Invitrogen Life Technologies/Thermo Fisher Scientific) stock solution (200 µM) was 1:500 diluted in cell culture medium shortly before application and added to the cell culture samples 1 h before the microcarrier co-incubation experiments were completed. After 1 h of MitoTracker Red dye incubation the cells were washed with PBS and fixed with PFA for further processing by immunofluorescence analysis as outlined before.

### 2.9. Confocal Microscopy (CLSM)

For confocal imaging, Leica TCS-SP8 was used. Images were recorded using a HC PL APO CS2 40x/1,30 objective with Leica Application Suite X (LAS X). For each sample (*n* = 3) five images of randomly selected microscopic fields were recorded. Every recording contained eight optical slices (z-stack: 6 µm) for maximum projection. DAPI was excited at 405 nm and detected at 417–467 nm. Alexa-488 was excited at 488 nm and detected at 506–530 nm. RITC and MitoTracker Red CMXRos, not applied in the same experiment, were both excited at 552 nm and detected at 601–667 nm and 650–706 nm, respectively. All parameters were selected regarding the avoidance of cross talk. The data were evaluated by visual approximation to allow comparison between cell lines. Thus, several criteria were defined: cells were counted by nucleus counting under consideration of the cytoskeleton (microtubule filaments). For uptake, microcarrier located within the microtubule filaments were counted. The deformation of the nucleus in presence of microcarriers is clearly visible as a distinct impression of the nucleus. Nucleus-near microcarriers are defined by nuclear contact of the microcarrier. Microtubuli-associated microcarriers show a distinct border caused by microtubules (often associated with a higher fluorescence intensity).

### 2.10. Statistical Analysis

Flux analysis data (technical replicates) of each measurement were normalized in the Agilent Wave Software (Agilent Seahorse Technologies) and exported as mean value to the Multi-File Seahorse XF Cell Energy Phenotype Test Report Generator. Standard deviation of a threefold approach (biological replicates) was then calculated with OriginPro 2017G. Data are shown as mean value with SD and were analysed by GraphPad software using repeated measures one-way ANOVA with Tukey’s post-hoc for flux analysis and *t*-test for quantification of cell and microcarrier parameters via immunofluorescence analysis. In experiments without drug application, control is defined as cells without application of microcarriers (no-carrier control). For the application of nocodazole, cell samples with microcarriers incubated in maintenance medium (maintenance control) or the solvent DMSO (solvent control) were employed as controls. * *p* < 0.05, ** *p* < 0.01, *** *p* < 0.001.

## 3. Results and Discussion

### 3.1. Influence of Microcarriers on Cellular Metabolic Potential

DDS are intended to have a broad use and as such need to target different cell types. Thus, we included in our study different cell lines with different basal metabolic activity (baseline phenotype) to assess the impact of microcarriers on cellular metabolism on a rather broad metabolic background.

Three human cell lines, two permanent epithelial cell lines (the adenocarcinomic alveolar basal A549, and human embryonic kidney HEK-293T/17(HEK)) and a cellular component of the inflammatory innate immune response (primary M1-like MΦ) besides two permanent animal cell lines (the mouse fibroblast NIH-3T3.CD4.CXCR4 [3T3] and epithelial African green monkey Vero cells). Extracellular flux analysis was used for assessment of mitochondrial respiration and glycolysis through simultaneous measurement of oxygen consumption rate (OCR) and extracellular acidification rate (ECAR), respectively. Furthermore, metabolic properties of a given cell type and the reliance on a more aerobic or anaerobic phenotype were addressed by the OCR/ECAR ratio calculated from basal OCR and ECAR. A low OCR/ECAR ratio reflects the reliance on glycolysis and the higher the value of the OCR/ECAR value, the higher the reliance on mitochondrial respiration [30]. Supplementary Materials Figure S1 reveals the rather low OCR/ECAR ratio of A549 in relation to the other cell lines, which in comparison to other cancer cell lines has a known high expression level of glycolytic enzymes and thus a high glycolysis level [31]. On the other hand, 3T3 have a high OCR/ECAR ratio reflecting a high rate of mitochondrial respiration and thus a reliance on oxidative phosphorylation. The implication of cell lines with different metabolic background in our study allows for assessment of the influence of microcarriers on cellular metabolism in a rather broad context.

Extracellular flux analysis was performed with the cell energy phenotype test kit, which enables characterization of the metabolic phenotype through co-injection of the ATPase inhibitor oligomycin and FCCP. Three measurement points of basal OCR and ECAR are followed by injection of these inhibitors, which refers to the stressed phenotype during extracellular flux measurement.

**Figure 1:**
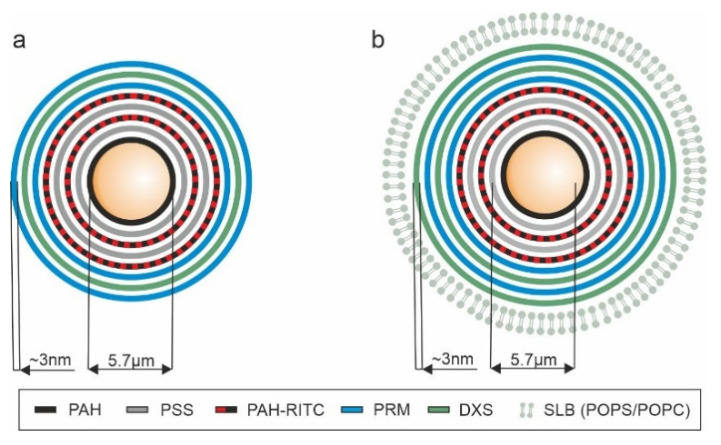
Illustration of the polymer and SLb microcarrier coating schemata. In (**a**) the polymer coating ending with negatively charged DXS (polymer microcarrier) is shown, (**b**) demonstrates microcarriers with a final SLB (POPS/POPC 1:1, POPS: anionic, POPC: zwitterionic) assembly on top of a positively charged polymer layer (SLB microcarriers).

LbL microcarriers with identical multilayer composition, but different surface characteristics (polymer or lipid layer) were applied to the four human and animal cell lines and M1-like MΦ as outlined before. LbL microcarriers consisted of a multilayer of PAH (PAH-RITC) and PSS as inner and PRM and DXS as biopolymer outer part (Figure 1). Both polymer pairs (PAH/PSS, PRM/DXS) are built up as a regular step-by-step multilayer as previous zeta potential measurements reveal [32,33]. As a final surface coverage, the carriers either terminated with a negatively charged polymer layer (DXS, named as polymer microcarriers, Figure 1a) or with a SLB (hereafter referred to as SLB microcarriers, Figure 1b). The main components of the lipid bilayer of SLB microcarriers consisted of zwitterionic phosphatidylcholine (POPC) and anionic phosphatidylserine (POPS) at equal molar ratios resulting in a negatively charged layer, the PE-PEG-biotin component, added at only 0.5 mol%, does not have an impact on the charge of the resulting lipid bilayer [25]. Thus, PRM was additionally assembled as an underlying layer of the SLB coating. With dominating micrometre-scaled core size, LbL microcarriers of the same size and surface charge were created to provide comparable conditions regarding cellular interaction, such that the subsequent investigations of uptake, intracellular pathway and cell metabolism only depend on the different surface functionalisation with a negatively charged polymer or SLB layer.

**Figure 2:**
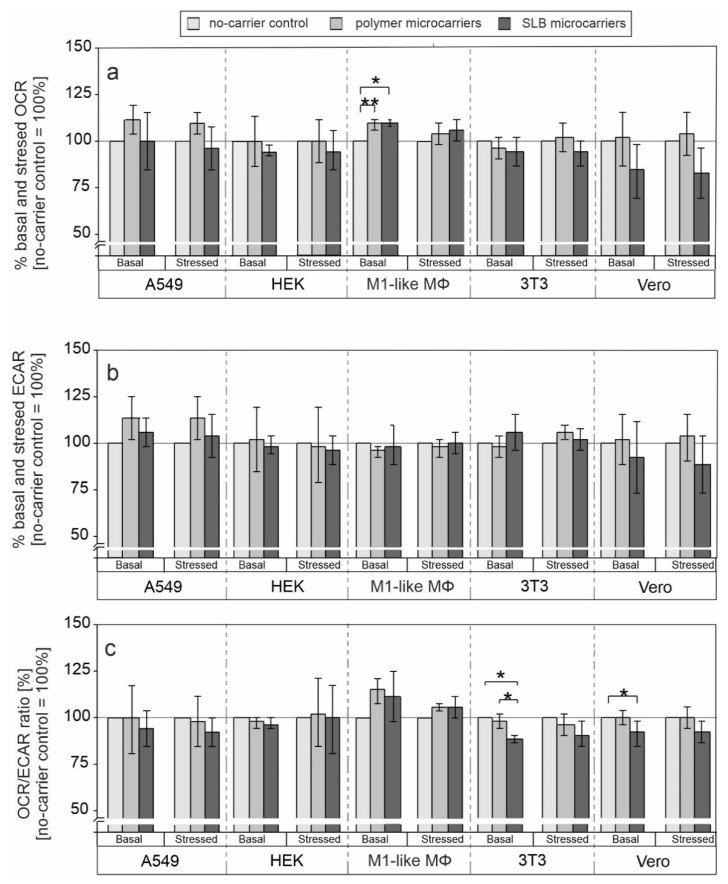
Influence of polymer and SLB microcarriers on mitochondrial respiration and glycolysis. The metabolic phenotype of the indicated cell lines was determined by extracellular flux analysis using the cell energy phenotype test kit. Basal and stressed (**a**) OCR and (**b**) ECAR values in addition to OCR/ECAR ratio (**c**) were obtained before and after injection of the inhibitors FCCP and oligomycin, respectively. Results are shown as percent change in relation to the untreated (no-microcarrier) control. The metabolic potential under basal and stressed (after injection of the inhibitors) conditions was calculated for indicated cell lines by the Energy Phenotype Test report generator software (*n* = 3, one-way ANOVA and Tukey’s post-hoc, * *p* < 0.05, ** *p* < 0.01).

Figure 2 shows basal and stressed (after co-injection of the inhibitors) OCR (Figure 2a) and ECAR (Figure 2b) and thus basal and stressed metabolic activity of various cell lines in response to internalization of microcarriers. Additionally, in the absence as well as presence of the microcarriers, basal and stressed OCR/ECAR ratio (Figure 2c) were calculated based on basal OCR and ECAR as this provides an indication whether application of microcarrier alters the cell-line specific metabolic activity (respiratory [aerobic] or more glycolytic [anaerobic]) [30].

Microcarriers were applied 24 h after plating to allow for cell adhesion except for a 2 h time frame for human M1-like MΦ as primary cell type. To reflect the in vitro differentiation process in the presence of GM-CSF, we refer to this MΦ type as M1-like MΦ. We set the focus on M1-like MΦ as they are pro-inflammatory and thus a potential target cell type for future DDS applications. Thereafter incubation with microcarriers was carried out for 16 h and at a micocarrier:cell ratio of 2:1. This time frame allowed for an optimal uptake and processing rate as revealed by our previous study, where uptake in different cell lines was shown to be completed at around 5–6 h [13]. While uptake for both, polymer and SLB microcarriers, progresses without loss of layer components, subsequent processing refers to passage of the endosomal-lysosomal compartment and the beginning of the disassembly of the lipid bilayer and polymer multilayer. In DDS applications, release of the microcarriers into cytoplasm and ongoing disassembly of the multilayer would be followed by drug release. Thus, this time frame was chosen for determination of the effect of microcarriers on cellular metabolism before an influence of the delivered cargo could occur.

Figure 2 shows basal and stressed OCR and ECAR values in the absence and presence of microcarriers. Values were calculated as fold change in reference to the control population in the absence of microcarriers (set as 100%). Figure S1 shows OCR and ECAR values for each cell line after normalization except for M1-like MΦ as non-proliferating and non-dividing primary cells. Normalization means that results were related to total protein concentration within the respective well measured as optical density (OD) (A549, HEK, Vero, 3T3) or based on equal cell numbers during plating (non-proliferative M1-like MΦ). In Figure 2 the control (cells without microcarriers) was set to 100% to demonstrate the effect of DDS on cells more clearly.

As can be observed for all applied cell lines (Figure 2 and Figure S1), the influence of both microcarrier types on mitochondrial respiration and glycolysis was generally rather low. However, cell type- and microcarrier functionalization-specific differences were noted. A significant increase in basal OCR after application of both microcarriers was only noted for M1-like MΦ (Figure 2a). This could be related to their function as a phagocytic cell type. M1-like MΦ are likely to take up microcarriers by phagocytosis, which represents a high energy-demanding process. This could result in the observed increase in OCR and thus in mitochondrial respiration. Vero cells were the only cell type for which a decrease in both OCR and ECAR occurred in comparison to the no-microcarrier control (Figure 2b). Moreover, this decrease was detected for both, basal and stressed ECAR by 7% and 11%, respectively. Additionally, the decrease in both, basal and stressed OCR was higher than the one detected in HEK and 3T3 cells (Figure 2). Except for human M1-like MΦ, after addition of SLB microcarriers a drop in basal OCR/ECAR ratio was noted for all cell lines (Figure 2c). Only Vero and 3T3 cells were positive for a significant drop in both, basal and stressed OCR/ECAR ratio.

**Figure 3:**
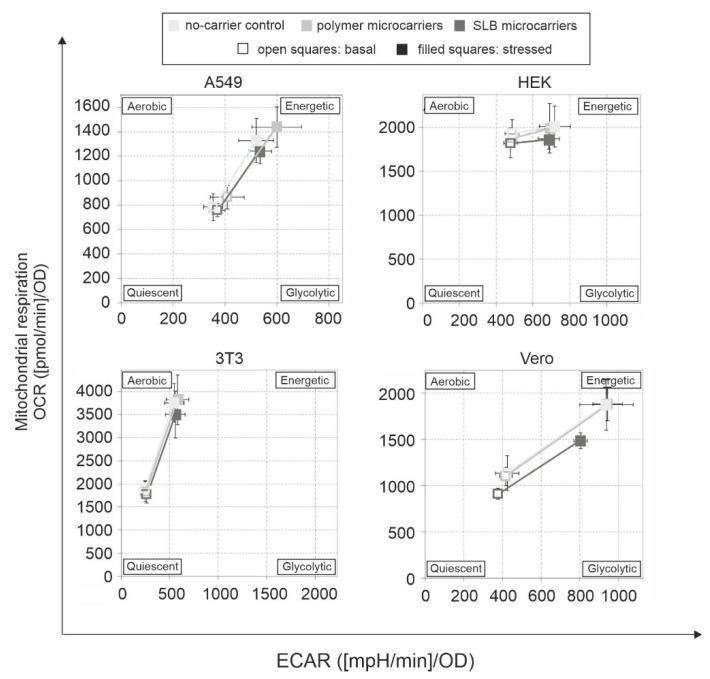
Cell energy phenotype for all examined cell lines incubated in the presence and absence of microcarriers. Energy maps were generated by the cell energy phenotype test kit based on OCR vs ECAR values obtained under basal and stressed conditions to reveal changes in energy maps after application of polymer and SLB microcarriers.

The cell energy phenotype test kit allows for assessment of the metabolic phenotype under basal and stressed conditions. Thus, it can be assessed whether a metabolic shift to a more aerobic or glycolytic activity occurred under a given experimental condition. Additionally, the increase of basal metabolic activity to the stressed metabolic activity after injection of the inhibitors reflects the metabolic potential of the cell under analysis. Figure 3 reveals a notable alteration in the energy phenotype only for SLB microcarriers and after incubation on Vero cells. Here, a decrease in both, the aerobic (mitochondrial) and glycolytic potential was present. Moreover, the metabolic potential was reduced (Figure 3).

In conclusion, while microcarriers exerted only a minor influence on cellular metabolism, one of the investigated cell lines, namely Vero, showed more pronounced alterations for these investigated parameters as revealed by a decrease in OCR and ECAR. Furthermore, due to a higher decrease in OCR compared to ECAR, a drop in OCR/ECAR ratio occurred.

### 3.2. Influence of the Lipid Bilayer on Association of Microcarriers with Microtubule Filaments and Their Perinuclear Localization

**Figure 4:**
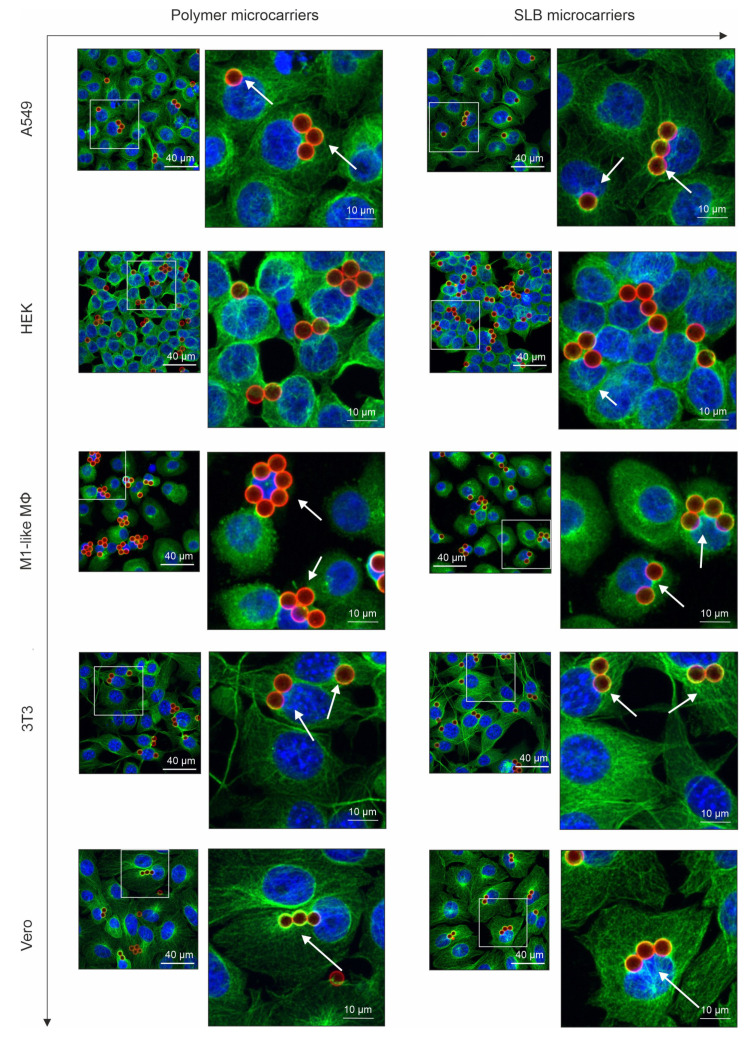
The association of microcarriers with microtubule filaments and their localization in a perinuclear area in different human and animal cell types and M1-like MΦ. Immunofluorescence analysis was performed after incubation of indicated cell types with polymer and SLB microcarriers for 16 h. The images were obtained after maximum intensity projections and the overlays show nuclei in blue (counterstained with DAPI), microtubule filaments in green (application of anti-α-tubulin antibodies) and microcarriers in red (labelling with RITC). The insets depict a higher magnification of indicated areas to reveal association of microcarriers with microtubule filaments (indicated by white arrows). Image size of the unzoomed images: 145 µm × 145 µm, zoom-ins: 58 µm × 58 µm.

In our previous study we identified an association of microtubule cytoskeleton with microcarriers [15]. Microtubules interact with both, mitochondria as it was shown for example for fission yeast [34] and with glycolytic enzymes [35]. Thus, we aimed at immunofluorescence analysis of microcarrier association with microtubule cytoskeleton as a possible factor contributing to our noted metabolic alterations (Figure 2). Qualitative analysis in Figure 4 reveals an association of both microcarrier types with microtubules, but at a cell type-dependent extent. In all examined cell lines microcarriers localize in the proximity of microtubule filaments, but with higher affinity of SLB microcarriers. Among the five cell types studied, Vero cells had the highest degree of co-localization of microcarriers with microtubule filaments and HEK the lowest. Moreover, microcarriers were in close proximity to the nucleus, either as single particles or even in clusters, which led to prominent deformations of nuclei. Perinuclear localization of microcarriers was especially pronounced in Vero cells. Moreover, in Vero cells localization of microcarriers in proximity to the nucleus and nuclear deformation events seem to be comparable between SLB and polymer microcarriers.

Figure S2 representatively shows a higher magnification of SLB microcarriers in Vero cells, which were localized in a perinuclear region and associated with nuclear deformation. Additionally, SLB microcarriers appear to be localized in the perinuclear region in a microtubule-dense area, which could represent the microtubule organizing centre (MTOC).

**Figure 5:**
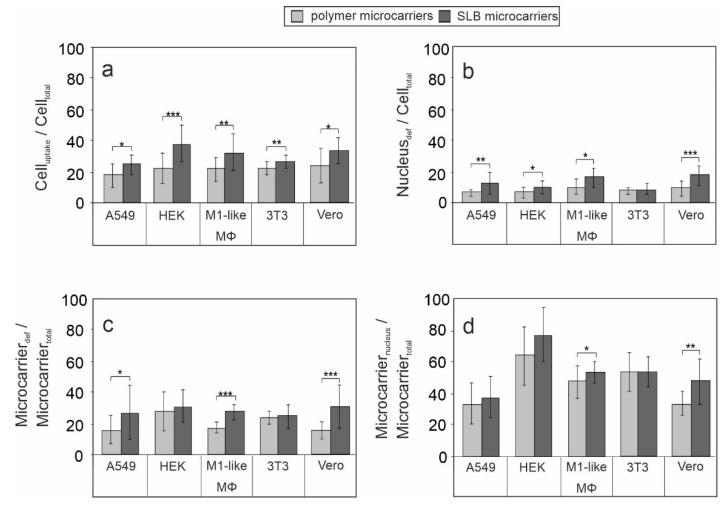
Determination of several parameters describing microcarrier interaction with cells. (**a**) shows internalized (Cell_uptake_) and (**b**) microcarrier-induced nuclear deformation (Nucleus_def_) to total cell number (Cell_total_); (**c**) microcarriers involved in nuclear deformation (Microcarrier_def_) and (**d**) nucleus-near, non-deforming microcarriers (Microcarrier_nucleus_) to total intracellular microcarrier number (Microcarrier_total_), (*n* = 3, five random microscopic fields were used for quantification *t*-test, * *p* < 0.05, ** *p* < 0.01, *** *p* < 0.001).

As a next step, we quantified intracellular localization and nuclear deformation events by the following four parameters to compare the five cell types and both microcarrier types (Figure 5).

First, the ratio of cells with at least one internalized carrier indicative for microcarrier uptake (Cell_uptake_) and total cell number (Cell_total_) was determined (Cell_uptake_/Cell_total_). This parameter reflects an estimate of the uptake capacity of the respective cell type. Figure 5a shows in comparison to polymer microcarriers a significantly higher internalization rate of SLB microcarriers on all five examined cell types. This indicates a cell line- and cell type-independent positive influence of lipid bilayer-based surface on the uptake of microcarriers. The highest uptake rate was achieved for SLB microcarriers on HEK cells (Figure 5a). Second, the ratio of cells with at least one microcarrier-induced nuclear deformation (Nucleus_def_) and total cell number (Cell_total_) was determined (Nucleus_def_/Cell_total_). Except for 3T3 cells, SLB microcarriers induced a significantly higher portion of deformed nuclei than polymer microcarriers (Figure 5b). The comparison of the five cell types indicates the highest level of deformed nuclei in M1-like MΦ and Vero cells. Third, the influence of the lipid bilayer-based surface on nuclear deformation events is especially indicated by the calculation of the ratio of internalized microcarriers which are involved in nuclei deformation (Microcarrier_def_) and the total carrier number in cells (Microcarrier_total_), the (Microcarrier_def_/Microcarrier_total_) ratio. On A549, M1-like MΦ and Vero cells the proportion of microcarrier-associated nuclear deformation events in comparison to total uptake events was significantly higher for SLB microcarriers in comparison to polymer microcarriers (Figure 5c). Fourth, the ratio of nucleus-near microcarriers (Microcarrier_nucleus_) compared to all internalized microcarriers (Microcarrier_total_), the (Microcarrier_nucleus_/Microcarrier_total_) ratio, was different within cell types, being the highest in HEK and the lowest in A549 cells (Figure 5d). Except for A549 cells, the Microcarrier_nucleus_/Microcarrier_total_ ratio was higher than the Microcarrier_def_/Microcarrier_total_ on the examined cell types. Thus, on A549 cells almost all microcarriers present in a perinuclear region result in nuclear deformation (Figure 5d). The lowest number of nuclear deformation events in relation to nuclear co-localization events was present in 3T3 cells (Figure 5d).

It is known, that cytoskeletal elements can naturally induce mechanical stress on the nucleus leading to nucleus confinement and deformation [36], an effect which is cell type dependent [37]. Additionally, mechanical deformation by externally added elements, such as functionalized microcarriers, seem not to lead to nucleus irritations, as no signs of apoptosis or necrosis could be found [13]. Thus, this system could then be adapted to transport active agents near the nucleus for specific applications, such as for nucleus-targeting cancer therapeutics [38].

In conclusion, among the addressed cell types of our study the positive influence of the lipid bilayer on microcarrier uptake und nuclear association was especially present in M1-like MΦ and Vero cells. On both cell types all applied positive parameters for intracellular microcarrier performance were significantly higher for SLB microcarriers than for polymer microcarriers.

### 3.3. Metabolic Alterations Coincide with Microcarrier Internalization and Relate to Mitochondrial Redistribution

**Figure 6:**
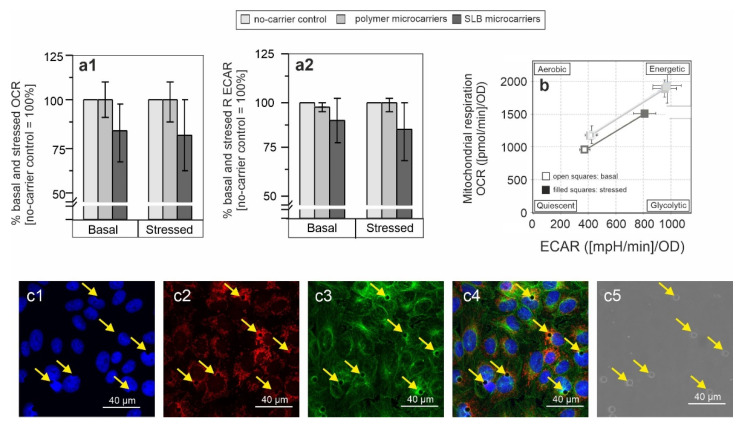
The early onset of SLB microcarrier-induced metabolic alterations in Vero cells was accompanied by their localization in close proximity to mitochondria. Vero cells were incubated with SLB microcarriers and processed for further analysis after 6 h of incubation. (**a**,**b**) The metabolic phenotype was determined by extracellular flux analysis using the cell energy phenotype test kit. Basal and stressed OCR (**a1**) and ECAR (**a2**) values were obtained before and after injection of the inhibitors FCCP and oligomycin, respectively. Normalized results are shown. (**b**) The metabolic potential under basal and stressed (after injection of the inhibitors) conditions was calculated after normalization by the energy phenotype test report generator software (n = 3, one-way ANOVA with Tukey’s post-hoc). (**c**) Immunofluorescence with antibodies against α-tubulin (shown in green) was performed after labelling of mitochondria with MitoTracker Red dye (shown in red). Nuclei are counterstained with DAPI and shown in blue, while SLB microcarriers are present in the visible light channel. Image size: 145 µm × 145 µm.

Microcarrier-induced metabolic alterations were especially present in Vero cells (Figure 2). Thus, we next focused on Vero cells to further address these metabolic alterations and to elucidate their time frame (Figure 6). For assessment of a shorter time frame after microcarrier uptake on Vero cells, the incubation of polymer and SLB microcarriers on Vero cells was reduced to 6 h, as from our previous work this time frame was shown to be sufficient to cover most uptake events [13,39].

Figure 6a shows, that already after an incubation for 6 h, SLB microcarriers induced a reduction in OCR and ECAR that was comparable to the 16 h incubation period (Figure 2). Similar to the 16 h incubation period, no effect was detected for polymer carriers (Figure 6a). Thus, it can be concluded, that indeed immediately after SLB microcarrier uptake the cells react with reduced metabolic activity, which is furthermore highlighted by the metabolic phenotype through the shift of mitochondrial respiration and glycolysis (Figure 6b). These findings led to the question, whether intracellular localization and association of microcarriers with specific cell compartments could be associated with the observed metabolic alterations. To address the reduction in OCR indicative for mitochondrial respiration, the mitochondria-specific dye MitoTracker Red dye was applied to Vero cells after 6 h of incubation with SLB microcarriers followed by immunofluorescence staining with anti-α-tubulin antibody for labelling of microtubule filaments (Figure 6c).

Figure 6c illustrates, that all SLB microcarriers were localized in close proximity to the nucleus and, as it was observed for the 16 h incubation period, in most cases massive deformation of the nucleus was detected. Moreover, SLB microcarriers appear to be surrounded by microtubule filaments and were often found in proximity to the MTOC. A weaker, but still existent correlation could be found with mitochondria, as SLB microcarriers were often found to be surrounded by mitochondria. Moreover, the distribution of mitochondria within Vero cells was slightly altered after incubation with SLB microcarriers to a more perinuclear clustering in proximity to the SLB microcarriers. Alterations of the distribution of mitochondria within cells are known to affect cellular metabolism [40] and such alterations can occur after infection with viruses and bacteria [41]. A zoom-in of these images is shown in Figure S3, illustrating the nucleus-near localization of SLB microcarriers and the resulting nuclear deformation besides an association with mitochondria. MitoTracker dyes are positively charged rosamine derivatives. Thus, we also tested for an unspecific labelling of microcarrier’s polymer network or lipid bilayer by the dye to rule out a microcarrier-associated fluorescence distribution. Figure S4 shows that after incubation of SLB microcarriers with MitoTracker Red dye no fluorescence signal was detectable on SLB microcarriers and therefore no unspecific labelling occurred. The localization of mitochondria in the proximity of microcarriers could result from their association with microtubule filaments, which are involved in the intracellular transport of mitochondria. In turn, the altered distribution of mitochondria could be a consequence of the association of SLB microcarriers with microtubules.

In conclusion, the reduced metabolic activity of Vero cells after addition of SLB microcarriers after incubation for 16 h was also present after 6 h. The reduced metabolic activity was not only paralleled by a close association with microtubule filaments, but also with mitochondria.

### 3.4. Assessment of the Association of SLB Microcarrier-Induced Metabolic Alterations with Microtubule Polymerization

**Figure 7:**
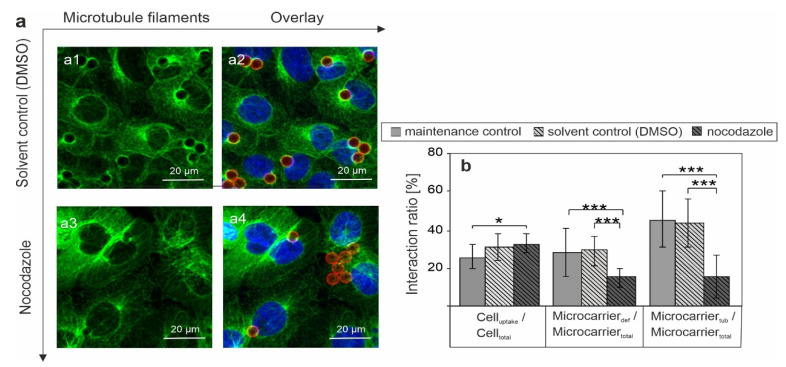
Immunofluorescence analysis of microtubule filaments in Vero cells after 4 h of incubation with SLB microcarriers and nocodazole (NOC). The solvent control (DMSO, **a1**,**a2**) or nocodazole (**a3**,**a4**) were added together with SLB microcarriers to Vero cells followed by replacement to maintenance medium after 4 h of incubation followed by an additional incubation time of 2 h. Nuclei were stained with DAPI (blue), microtubule filaments with anti-α-tubulin (green), and microcarriers with RITC (red). In (**b**) SLB microcarriers were added in maintenance medium (maintenance control), in the solvent control (solvent DMSO) or nocodazole medium to Vero cells. Immunofluorescence samples were subjected to quantification of cell and microcarrier properties. Data are presented as the ratio of cells with at least one internalized microcarrier to the total number of cells (Cell_uptake_/Cell_total_), the ratio of microcarrier-associated with deformed nuclei to total number of microcarriers (Microcarrier_def_/Microcarrier_total_, and the ratio of internalised microcarrier, which are associated with microtubule filaments (Microcarrier_tub_/Microcarrier_total_), (*n* = 3, five random microscopic fields were used for quantification *t*-test, * *p* < 0.05, *** *p* < 0.001). Image size: 75 µm × 75 µm.

To address the possible correlation between microtubule association (up to localization in proximity to the MTOC) of SLB microcarriers and their impact on cellular metabolism, microtubule network organisation of Vero cells was altered through addition of 10 µM nocodazole. Nocodazole interferes with microtubule polymerization. While this irritation is reversible and requires only short incubation times, a long-term application of nocodazole could affect cell viability and in this regard cellular metabolism. Thus, a short but sufficient incubation time-frame is required. As an experimental approach, nocodazole and SLB microcarriers were simultaneously applied to Vero cells, and after 4 h of incubation, nocodazole or the solvent DMSO (the solvent control) were replaced by maintenance medium for another incubation of 2 h, which covers the 6 h incubation time frame presented in Figure 6. The application of DAPI as nuclear counterstain was combined with staining of the microtubule network with anti-α-tubulin and a RITC label to localize the microcarriers. This labelling approach allowed for qualitative and quantitative analysis of microcarrier distribution in nocodazole-treated Vero cells in comparison to the solvent control, the results are shown in Figure 7.

Compared to previous experiments, the solvent control now included Vero cells with DMSO in the concentration present in the nocodazole application. In the solvent control SLB microcarriers were associated with microtubule filaments and localized to the MTOC in close proximity to nuclei, which led to nuclear deformation (Figure 7(a1,a2)). However, and most importantly, these observations were strongly reduced after nocodazole application (Figure 7(a3,a4)). While still associated with the microtubule network, the microcarriers seemed to be more homogeneously distributed over the cytoplasm and much less SLB microcarriers were found to be co-localized with MTOC, and nuclear deformation was reduced accordingly. Figure 7b shows the quantification of these observations on nocodazole-associated alterations of the intracellular localization of SLB microcarriers. This included the comparison of SLB microcarriers in maintenance medium with SLB microcarriers in the solvent control. While solvent control treatment was similar to the incubation in maintenance medium, nocodazole application induced specific effects in SLB carrier localization. In particular, Cell_uptake_/Cell_tot_ remained almost constant, just with a slight, but not significant increase in SLB microcarrier uptake in the presence of the solvent control as well as nocodazole as compared to the maintenance control. However, a prominent and significant reduction (by 49%) in SLB microcarrier-induced nuclear deformation (Microcarrier_def_/Microcarrier_total_) occurred after addition of nocodazole, indicating that microtubule filaments are involved in the intracellular transport of SLB microcarriers to a perinuclear region. Moreover, in comparison to the solvent control the association of SLB microcarriers with microtubule filaments was significantly reduced after incubation with nocodazole. Furthermore, for the analysis of the application of nocodazole we employed an additional parameter for quantification, the ratio of microcarrier number associated with microtubules to total number of microcarriers. Here, the ratio Microcarrier_tub_/Microcarrier_total_ was reduced after nocodazole treatment by 66% in comparison to the maintenance control and by 64% in comparison to the solvent control. These results clearly demonstrate that SLB microcarriers are indeed associated with the microtubule network, a correlation which appears to contribute to their localization in a perinuclear region and as such possibly to nuclear deformation events.

**Figure 8:**
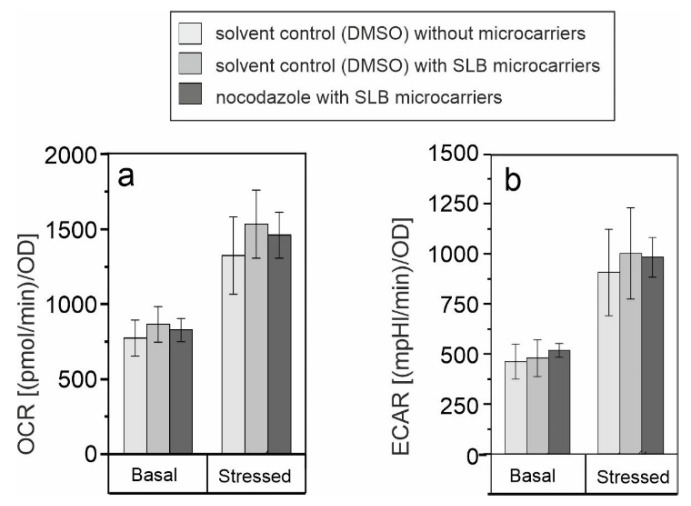
Extracellular flux analysis with the cell energy phenotype test kit was performed to measure basal and stressed (**a**) OCR (mitochondrial respiration) and (**b**) ECAR (glycolysis) in Vero cells under the shown conditions. Measurements were normalized to total protein content. (n = 3, one-way ANOVA with Tukey’s post-hoc, significance versus the control (Vero in the solvent control and in the absence of microcarriers).

The effect of the microtubule polymerization inhibitor nocodazole on SLB microcarrier-associated metabolic alterations was then investigated through assessment of basal and stressed OCR and ECAR through application of the cell energy phenotype test kit. Figure 8 reveals a comparable impact of SLB microcarriers in the solvent control and SLB microcarriers in nocodazole on basal and stressed OCR (Figure 8a) and ECAR (Figure 8b) and as such on cellular metabolic activity.

However, in comparison to the maintenance control, stressed OCR was only slightly increased after incubation with SLB microcarriers in both, the solvent control and in the nocodazole samples. The metabolic activity of Vero cells in the presence of microcarriers was comparable between the solvent control and nocodazole. No significant differences were detected. The use of the solvent DMSO itself could alter the performance and viability of cells [42], which strengthens the need for a comparison to the maintenance control lacking DMSO. Furthermore, the impact of the solvent DMSO on the lipid bilayer within SLB microcarriers also needs to be taken into consideration in the interpretation of our data set, since lipid bilayer damage can be induced [43].

In summary, the addition of the microtubule polymerization inhibitor nocodazole during the incubation of SLB microcarriers on Vero cells reduced their perinuclear localization and microtubule association without a notable alteration of SLB microcarrier-induced effects on cellular metabolism.

## 4. Conclusions

As lipid-based DDSs gain increasing attention as transport vehicles for immunotherapeutic agents, the transport and effects of the carrier itself on cellular metabolism remain an interesting issue. Here we built up Layer-by-Layer coated microcarriers and functionalised their surface either with a phospholipid-based supported lipid bilayer (SLB; POPS/POPC, including a PE-PEG-biotin anchor) or with a plain, negatively charged polymer layer (polymer; dextran sulphate) in order to study the impact of such a lipid shell on cells. As markers for metabolic activity, mitochondrial respiration and glycolysis were investigated accompanied by immunofluorescence to get information about the microcarrier association with microtubule filaments, the nucleus and mitochondria, while several cell types with different metabolic activity were employed.

In general, SLB- as well as polymer-coated microcarriers appear to have only a minor influence on cellular metabolism. Here, only Vero cells stand out with slightly decreased mitochondrial respiration and glycolysis. While SLB-coated microcarriers show indeed differences to polymer microcarriers in all investigated cell types, they are pronounced in Vero, but also in M1-like MΦ: SLB microcarriers undergo a higher uptake rate, a faster and intracellular movement and stronger association with nuclei up to pronounced nucleus deformations. Additionally, the reduced metabolic activity in Vero cells correlates with SLB microcarrier association with microtubule filaments, and in particular with the perinuclear MTOC, as well as with mitochondria. The application of nocodazole as a microtubule polymerization inhibitor then demonstrated that microtubule filaments appear to be the driving force for the microcarrier transport within the cell and their final localization in close proximity to the nucleus. Such a process now opens up several options to develop strategies to use these parameters for the development of nucleus-associated DDSs in nano- and micrometre size.

## Data Availability

Data are available on request.

## Supplementary Materials

The following are available online at https://www.mdpi.com/article/10.3390/pharmaceutics13091441/s1, Figure S1: Extracellular flux analysis of metabolic properties of all given cell types after 16 h incubation, Figure S2: Localization of SLB microcarriers in Vero cells. Figure S3: SLB microcarriers (transmission image, c) in Vero cells with mitochondria (red), microtubule filaments (green) and nucleus (blue) staining, as seen in overlay (b). Figure S4: Intracellular control staining of SLB microcarriers with mitochondria-specific dye MitoTracker Red.
