# Supplementary material for: The Metabolic Response of Various Cell Lines to Microtubule-Driven Uptake of Lipid- and Polymer-Coated Layer-by-Layer Microcarriers
Source: Pharmaceutics. 2021 Sep 10;13(9):1441. doi: 10.3390/pharmaceutics13091441 (PMC8465159; doi:10.3390/pharmaceutics13091441)
Supplement: Supplementary file 1 [file pharmaceutics-13-01441-s001.zip › pharmaceutics-1336503-supplementary.pdf]

pharmaceutics

MDPI

Supplementary Material

The Metabolic Response of Various Cell Lines to Microtubule-Driven Uptake of Lipid- and Polymer-Coated Layer-by-Layer Microcarriers

Claudia Claus [1,†,*], Robert Fritz [2,†], Erik Schilling [3] and Uta Reibetanz [2,*]

1 Institute of Medical Microbiology and Virology, Faculty of Medicine, University of Leipzig, Johannisallee 30, 04103 Leipzig, Germany
2 Institute for Medical Physics and Biophysics, Faculty of Medicine, University of Leipzig, Härtelstrasse 16-18, 04107 Leipzig, Germany; fritz_robert@gmx.net
3 Institute of Clinical Immunology, Faculty of Medicine, University of Leipzig, Johannisallee 30, 04103 Leipzig, Germany; erik.schilling@medizin.uni-leipzig.de
* Correspondence: claudia.claus@medizin.uni-leipzig.de (C.C.); uta.reibetanz@medizin.uni-leipzig.de (U.R.); Tel.: +49-341-9714321 (C.C.); +49-341-9715703 (U.R.)
† These authors contributed equally to this manuscript

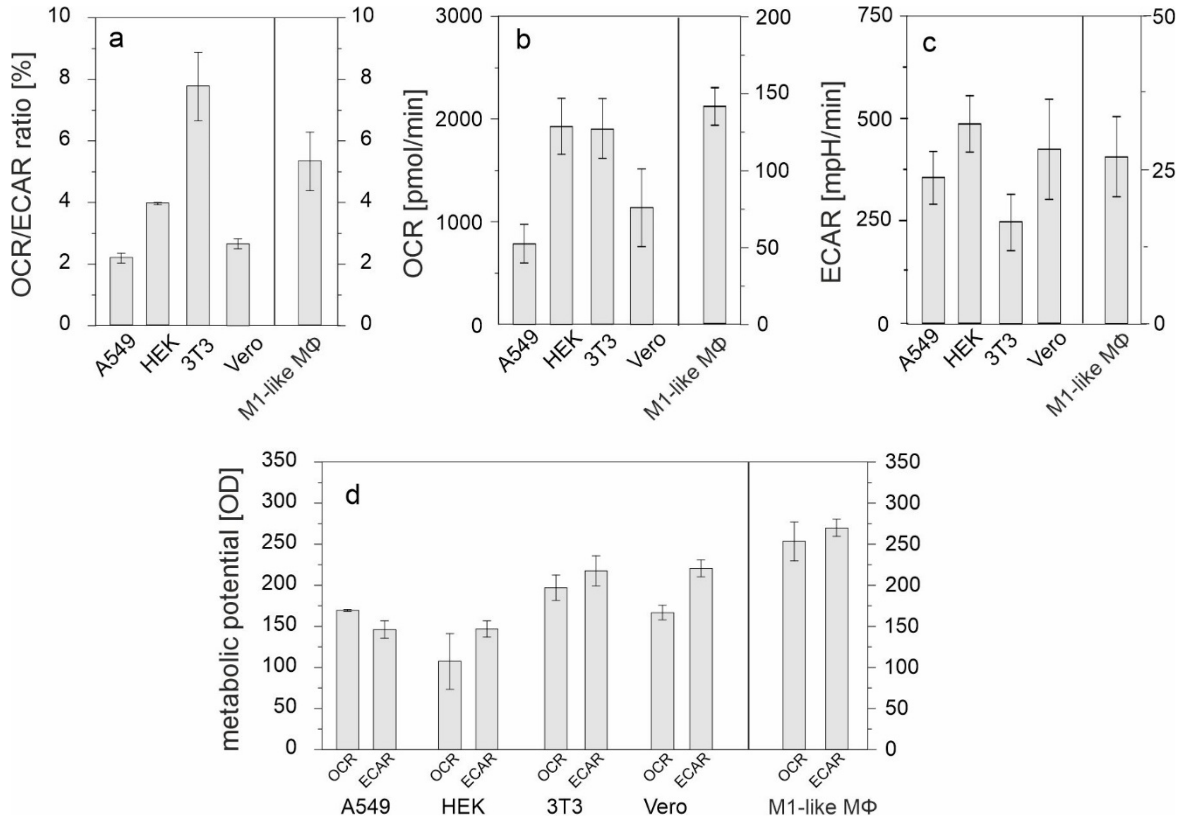

Figure S1. Extracellular flux analysis of metabolic properties of all given cell types after 16 h incubation. In (**a**) OCR/ECAR ratio, (**b**) OCR and (**c**) ECAR are presented after flux analysis of A549, HEK, 3T3, Vero and M1-like MΦ without microcarrier application. In (**d**) metabolic potential regarding increase in stressed OCR and ECAR over basal OCR and ECAR, respectively, is shown. A549, HEK, 3T3 and Vero measurements are normalized on protein concentration (OD) using a Bradford assay, while M1-like MΦ were used as previously defined based on equal cell numbers during plating.

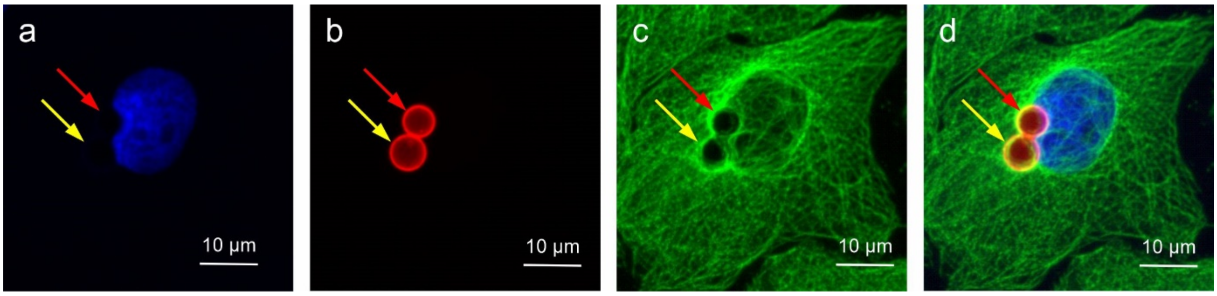

Figure S2. Localization of SLB microcarriers in Vero cells. SLB microcarriers (red, (**b**)) were mostly localized in perinuclear regions, where they were involved in nuclear deformations (blue, (**a**)). Additionally, they were associated with microtubule-dense areas (green, (**c**)), which appear to represent the microtubule organizing center (MTOC, dense green structure with high intensity). Overlay is shown in (**d**). Image size: 50µm × 50µm.

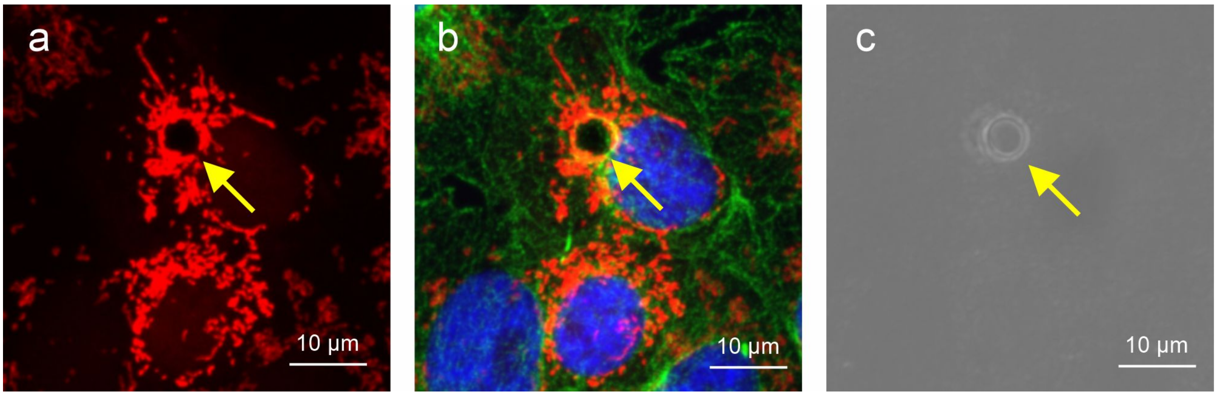

Figure S3. Analysis of SLB microcarriers in Vero cells after incubation with SLB microcarriers for 6 h. Mitochondria are shown in red (**a**) and in the overlay (**b**), which also includes microtubule filaments (green) and nuclei (blue). SLB microcarriers are shown in the transmission image, (**c**) SLB microcarriers appeared in perinuclear localization and in association with strong nuclear deformations and mitochondria. Image size: 50µm × 50µm.

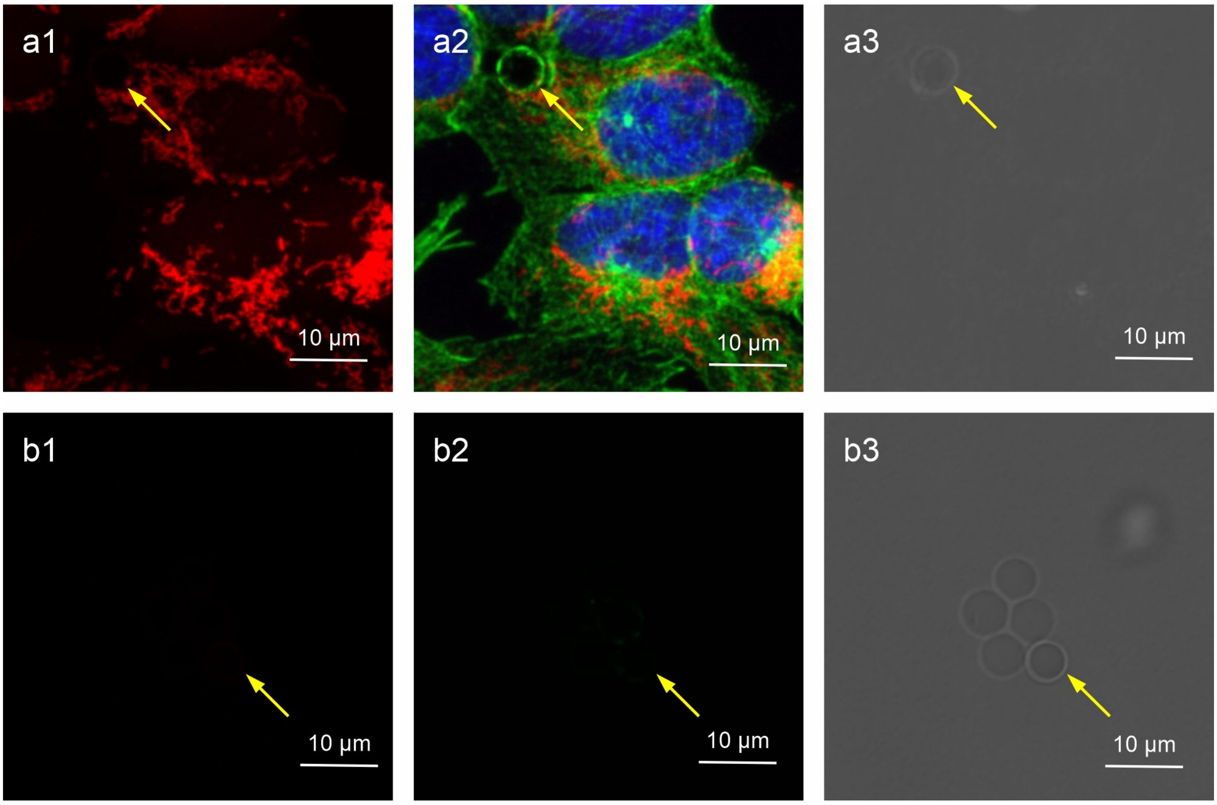

Figure S4. Intracellular staining of mitochondria with MitoTracker Red dye in Vero cells after incubation with SLB microcarrier for 6 h. The application of MitoTracker dyes as positively charged rosamine derivatives raises the possibility of co-staining of the lipid membrane or polymer network of the microcarriers. To address this possibility, unlabelled SLB microcarriers were applied to Vero cells for 6 h, followed by incubation with MitoTracker Red (red: mitochondria), DNA counterstain (blue: nucleus) and immunofluorescence analysis with anti-tubulin antibodies (green: microtubule) with overlays in (a2, b2); transmission: SLB microcarriers (a3, b3)). Intracellular microcarriers (**a**) and extracellular microcarriers (**b**) were analyzed. Within a representative cell (**a**), unlabelled SLB microcarriers appear with a green shell. No MitoTracker Red labelling of microcarriers was observed. In extracellular space (**b**), unlabelled SLB microcarrier were not stained by MitoTracker Red or anti-◎-tubulin antibodies. Thus, intracellular green shell of SLB microcarriers was caused solely by its association with stained microtubule filaments. Image size: 50µm x 50µm. .
